# Analysis of Genetic Diversity in Romanian Carpatina Goats Using SNP Genotyping Data

**DOI:** 10.3390/ani14040560

**Published:** 2024-02-07

**Authors:** Bogdan Alin Vlaic, Augustin Vlaic, Isa-Rita Russo, Licia Colli, Michael William Bruford, Antonia Odagiu, Pablo Orozco-terWengel, CLIMGEN Consortium

**Affiliations:** 1 Department of Animal Breeding, Faculty of Animal Science and Biotechnologies, University of Agricultural Sciences and Veterinary Medicine Cluj-Napoca, Mănăștur Street 3–5, 400372 Cluj-Napoca, Romania; bogdan.vlaic@usamvcluj.ro; 2 Faculty of Animal Science and Biotechnologies, University of Agricultural Sciences and Veterinary Medicine Cluj-Napoca, Mănăștur Street 3–5, 400372 Cluj-Napoca, Romania; vlaic.augustin@gmail.com; 3 Cardiff School of Biosciences, Cardiff University, The Sir Martin Evans Building, Museum Avenue, Cardiff CF10 3AX, Wales, UK; russoim@cardiff.ac.uk (I.-R.R.); brufordmw@cardiff.ac.uk (M.W.B.); 4 Dipartimento di Scienze Animali, della Nutrizione e degli Alimenti (DIANA), BioDNA Centro di ricerca sulla Biodiversità e sul DNA Antico, Facoltà di Scienze Agrarie, Alimentari e Ambientali, Università Cattolica del Sacro Cuore, Via Emilia Parmense n. 84, 29122 Piacenza, PC, Italy; licia.colli@unicatt.it; 5 Faculty of Agriculture, University of Agricultural Sciences and Veterinary Medicine Cluj-Napoca, Mănăștur Street 3–5, 400372 Cluj-Napoca, Romania

**Keywords:** goat, *Capra hircus*, diversity, breeding, SNP

## Abstract

**Simple Summary:**

The Carpatina goat breed from Romania is very well adapted to a range of contrasting and very different environments compared to highly specialized breeds. This adaptability makes them an important source of genetic variability for future breeding programs. To improve breeding strategies, it is necessary to evaluate the genetic diversity of the Carpatina breed. The aim of this study was therefore to investigate genetic diversity and inbreeding of old native Carpatina goat populations from different geographical areas in Romania using single nucleotide polymorphism (SNP) data for 67 individuals. Diversity, measured by average expected heterozygosity (*H_E_*), was 0.418, and average observed heterozygosity (*H_o_*) was 0.420. Linkage disequilibrium (LD) estimation showed that the average r^2^ value was 0.32. The effective population size (*N_e_*) estimate 13 generations ago ranged between 98 and 185. These results based on SNP data are very useful for the application of proper management and for designing goat breeding programs.

**Abstract:**

Animal husbandry is one of man’s oldest occupations. It began with the domestication of animals and developed continuously, in parallel with the evolution of human society. The selection and improvement of goats in Romania was not a clearly defined objective until around 1980. In recent years, with the increasing economic value given to goats, breeding programs are becoming established. In Romania, a few goat genetic studies using microsatellites and mtDNA have been carried out; however, a systematic characterization of the country’s goat genomic resources remains missing. In this study, we analyzed the genetic variability of Carpatina goats from four distinct geographical areas (northern, north-eastern, eastern and southern Romania), using the Illumina OvineSNP60 (RefSeq ARS1) high-density chip for 67 goats. Heterozygosity values, inbreeding coefficients and effective population size across all autosomes were calculated for those populations that inhabit high- and low-altitude and high- and low-temperature environments. Diversity, as measured by expected heterozygosity (*H_E_*), ranged from 0.413 in the group from a low-temperature environment to 0.420 in the group from a high-temperature environment. Within studied groups, the HT (high temperature) goats were the only group with a positive but low average inbreeding coefficient value, which was 0.009. After quality control (QC) analysis, 46,965 SNPs remained for analysis (MAF < 0.01). LD was calculated for each chromosome separately. The *N_e_* has been declining since the time of domestication, having recently reached 123, 125, 185 and 92 for the HA (high altitude), LA (low altitude), HT (high temperature) and LT (low temperature) group, respectively. Our study revealed a low impact of inbreeding in the Carpatina population, and the *N_e_* trend also indicated a steep decline in the last hundred years. These results will contribute to the genetic improvement of the Carpatina breed.

## 1. Introduction

The Romanian Carpatina goat was formed in mountainous and hilly areas of the Carpathian Mountains and belongs to the southern European group of goats [1]. Known as Karpacka, the Carpatina breed is native to Romania, where individuals can exhibit a variety of colors, whereas outside of their native range, i.e., in Poland, the breed tends to be uniformly white [2]. Historically, Carpatina goats were bred across a wide range of environments in Romania and have been under low levels of human-mediated selective pressure, which led to high phenotypic variability, possibly due to zonal adaptation reflecting contrasting environments in Romania (e.g., the colder upper Carpathian Mountains vs. the warmer southern lowlands). Romanian goat owners rear Carpatina all over the country, on small and large goat farms, mostly following extensive farming approaches. Contrastingly, subsistence farmers usually have 1–3 goats, maintained in flocks with goats of other owners, grazing around villages. According to the Eurostat 2022 reports, the Romanian total stock of goats was around 1.51 million animals (https://ec.europa.eu/eurostat/databrowser/view/apro_mt_lsgoat/default/table?lang=en, accessed on 24 March 2023), with Carpatina representing over 90% of the total [3,4].

The Carpatina goat is regarded as low-performing, but the breed is characterized by good adaptation and resistance to environmental conditions. Several studies showed modest production levels: milk yield estimates of 220–350 kg/lactation, litter size of 130–160% and growth rates in kids between 90 and 110 g/day [5,6,7]. Due to the perceived low milk production levels, goat owners have increased their flocks with exotic breeds known for their high milk yields, e.g., Saanen or French Alpine. Carpatina is a multipurpose breed, with its main traits being good zonal adaptability, medium size, diversity of dairy products, large productivity variability, an outer appearance specific to primitive animals, fine-robust body, lively behavior, high mobility and agility, adaptation to climate stress, mixed haired, multicolored and presence of twisted horns [2,8]. Due to its reasonable reproductive and productive performances, it is desirable to improve Carpatina’s production efficiency to make the breed more competitive with industrial breeds and to ensure Carpatina’s genetic integrity and self-sustainability. Although several goat breeds have already been characterized genetically [9,10,11,12,13,14], at the national level few studies have included Carpatina in genetic analyses, which have been limited to a handful of traditional genetic markers [4,7,9] and a study on worldwide goats using SNP array data that included 14 Carpatina goats [9]. Even though the Carpatina goat is well known for its robustness, resistance and adaptability, factors such as environmental stresses derived from climate change, or genetic erosion through hybridization with other breeds, threaten the breed’s future.

Characterizing the genetic variation in a species is critical for management practices to be adequately informed, so that the genetic variation is preserved and prevented from eroding. Capturing information on genetic variation can be achieved in several ways, by studying genetic markers and estimating parameters such as the observed and expected heterozygosity. The inbreeding coefficient provides further information regarding the breeding of consanguineous individuals in a population, and thus provides additional relevant information to be considered in breeding. However, an often neglected but critical parameter is the effective population size (*N_e_*). This population genetic parameter reflects genetic variation, genetic drift and linkage disequilibrium in a population [15]. In livestock, the decrease of the effective population size is usually largely due to intensive reproduction and genetic management practices, e.g., increased inbreeding through the use of small numbers of bucks for breeding [16]. The management of goat genetic resources involves certain priority actions, i.e., to establish a coherent and comprehensive breeding program [17] and to minimize the substitution of native breeds by exotic breeds. According to Carolino et al. [18], genetic improvement of goat populations is a key factor in future livestock production, making them resistant to climate change consequences while maintaining high-quality productions.

In this context, we aimed to characterize the genome-wide genetic variation of Romanian Carpatina goats and determine whether there were marked differences between groups of Carpatina that are historically kept in different Romanian regions. Our work represents the first large-scale genomic characterization of Carpatina and provides a baseline against which breeders can measure the progress of breeding strategies as well as their compliance with the goals defined by the Global Biodiversity Framework that was agreed upon in December 2022.

## 2. Materials and Methods

### 2.1. Goat Sample Collection and Experimental Design

**Figure 1:**
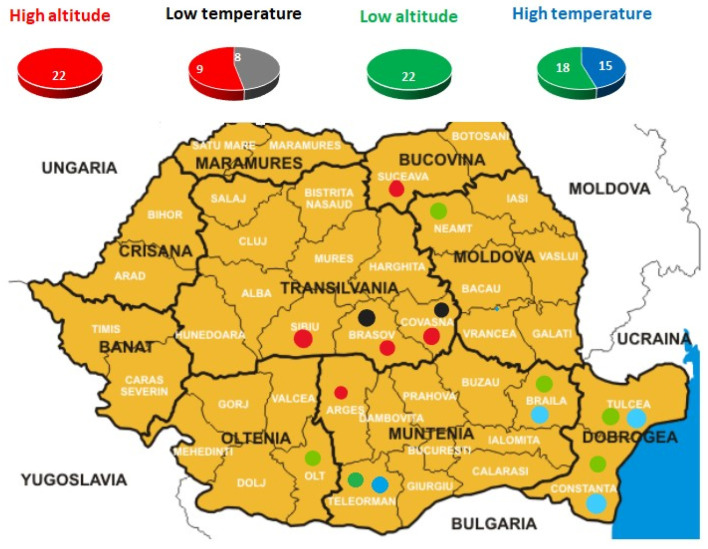
Geographical locations of the sampled Carpatina goats.

All animal work and experimental design were approved by the Faculty of Animal Sciences and Biotechnologies, University of Agricultural Sciences and Veterinary Medicine, Cluj-Napoca, Romania, with the required ethical approval. A total of 67 goats were sampled in contrasting climatic environments across Romania, with sample sizes per group of animals ranging between 17 and 33, which correspond to between 94% and 97% chance of including those groups’ complete genealogy, including their most recent common ancestor [19]. Sampling focused on the following counties due to their environmental differences: Suceava, Sibiu, Covasna, Brasov, Arges, Neamt, Braila, Tulcea, Constanta, Olt and Teleorman (Figure 1). For each animal, blood was collected from the jugular vein by an authorized veterinarian and stored in 20 mL EDTA tubes.

For each region, historic climate recordings (environmental maps) were obtained from the National Report on the State of Environment in Romania (ANPM 2013–2014, available online in Romanian at: http://www.anpm.ro/raport-de-mediu, accessed on 24 March 2023). The environmental variables recorded for each sampling site were high altitude (HA), low altitude (LA), high temperature (HT) and low temperature (LT). It should be mentioned that some individuals correspond to several climate environments (e.g., an individual can be found in a HA site that is also of LT; Table 1). GPS coordinates were determined for each sampling area. The goats were selected on the basis of information received from the breeders, in order to avoid sampling from related individuals.

### 2.2. Genotyping and Quality Control

DNA extractions and genotyping were conducted at the AGROTIS S.R.L-Laboratorio Genetica e Servizi (Cremona, Italy) using the Illumina ovineSNP60 Bead chip (Illumina, San Diego, CA, USA). Illumina Bead Studio Data Analysis Software (V2.0) was used to extract all genotype calls from the raw dataset. SNP data quality control was performed using the PLINK v1.9 software [20]. We removed individuals with 10% or more missing data and SNPs with a call rate of 95% or less, as well as SNPs with a MAF of less than 0.01. Only autosomes were kept for further analysis. Lastly, SNPs not in Hardy–Weinberg Equilibrium (HWE *p*-value < 0.001) were removed.

### 2.3. Genetic Diversity

PLINK v1.9 was used for the estimation of minor allele frequency (MAF), mean expected (*H_E_*) and observed (*H_o_*) heterozygosity, average relatedness (PI_HAT) and average individual inbreeding coefficients (*F_IS_*) based on the observed versus expected number of homozygous genotypes, using the formula:

*F_IS_ = (O_i_ − E_i_)/(L_i_ − E_i_)*

where *F_IS_* is the inbreeding coefficient, *O_i_* is the observed autosomal homozygous genotype counts, *E_i_* is the expected autosomal homozygous genotype counts, and *L_i_* is the total number of observations. *H_E_* and *H_o_* were estimated using—hardy flag, while the inbreeding coefficients (*F_IS_*), MAF and average relatedness (PI_HAT) were estimated using—het, —freq and—genome commands. Pairwise *F_st_* values between the groups of goats defined by temperature or altitude were estimated with Arlequin v. 3.5.2.2 [21]. An analysis of molecular variance (AMOVA) was carried out in Arlequin to analyze the partitioning of genetic variation at different hierarchical levels, i.e., individual, within-group and between groups.

### 2.4. Linkage Disequilibrium (LD) and Effective Population Size (N_e_)

We used PLINK v.1.9 software to estimate LD measured as r^2^. The r^2^ parameter is considered the squared correlation coefficient (r) between allele frequencies at two loci. The parameters—ld-window-kb 100 k and—ld-window-r^2^ set to zero were used to estimate LD between SNPs up to 100,000 bp apart from each other in the same chromosome and recording all r^2^ values in the range of zero to one. LD was estimated in each group separately.

The effective population size (*N_e_*) is a parameter that quantifies the turnover in genetic variation due to the effect of genetic drift in a population [22]. This parameter was estimated using the SNeP software version 1.1, using Sved’s [23] formula: *E*(r^2^) = (1 + 4*N_e_c*)^−1^, where *N_e_* is the effective population size, *c* is the genetic distance between two markers (expressed in Morgans), and *E*(r^2^) is the expected r^2^ for a given distance *c*. Time points representing the number of generations in the past were estimated as T = 1/2*c* [24], with a generation interval of ~4 years [25].

## 3. Results

### 3.1. Genetic Diversity

Across the 67 samples collected, 95.9% SNPs were retained for analyses and no individual was dropped. The average MAFs across SNPs ranged from 0.328 (LT) to 0.335 (HT) with an overall mean of 0.332. The observed and expected heterozygosity for the total dataset were identical (0.418), and the estimates were also very close for the two summary statistics in each of the four groups (Table 2). The small difference in observed versus expected heterozygosity suggests that inbreeding has played little or no role in shaping the genetic variation in Carpatina. Consistent with these expectations, the inbreeding coefficients estimated for each group of the total dataset were not significantly different from zero (*p*-value > 0.05). Overall, the largest proportion of genetic variation was found within individuals (~98.22%, while less than 1.23% was found within groups and 0.57% was found between groups). The results from the AMOVA are reflected in the pairwise *F_st_* values which are close to zero (Table 3). For some pairwise *F_st_* values, the *p*-value resulted after significant False Discovery Rate (FDR) correction (*p*-value < 0.02; [26]); however, the *F_st_* values indicated a divergence in allele frequencies between the groups (0.7% or less).

### 3.2. Linkage Disequilibrium (LD) and Effective Population Size (N_e_)

As expected, the number of SNPs was proportional to chromosome size, with the highest number of SNPs found on chromosome 1 and the lowest number found on chromosome 25 (Table S1). Average Linkage Disequilibrium (r^2^) ranged from 0.0469 (LA) to 0.0782 (LT) with an overall mean of 0.0621 for all four groups. For the HA group, the highest average LD was found on chromosome 12 (0.0715) and the lowest on chromosome 9 (0.0593). The highest and lowest values of r^2^ for both the LA and LT groups were found on chromosome 12 (0.0710 and 0.0869) and chromosome 29 (0.0559 and 0.0736). The HT group presented the highest LD value on chromosome 11 (0.0617) and the lowest value on chromosome 28 (0.0411).

**Figure 2:**
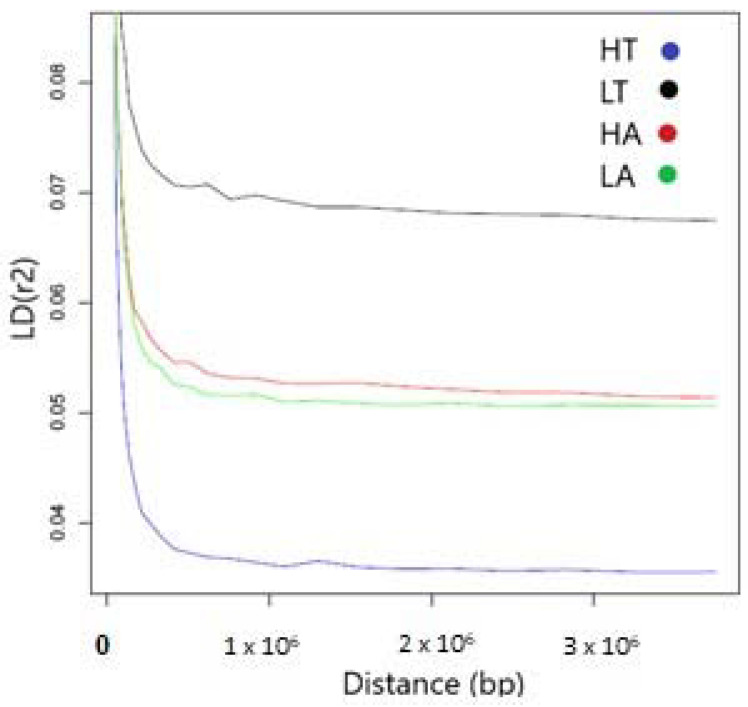
LD decay (r^2^) for the Carpatina groups.

The four populations showed the same type of Linkage Disequilibrium decay curve, with a steep decline in LD within the first 100,000 bp between two markers and reaching a plateau when the average distance between pairs of markers was ~500,000 or more. Although the four populations showed similar LD decay patterns, overall LT presented a higher LD curve, reflecting higher r^2^ values across the genome. Contrastingly, HT presented overall lower r^2^ values (Figure 2).

**Figure 3:**
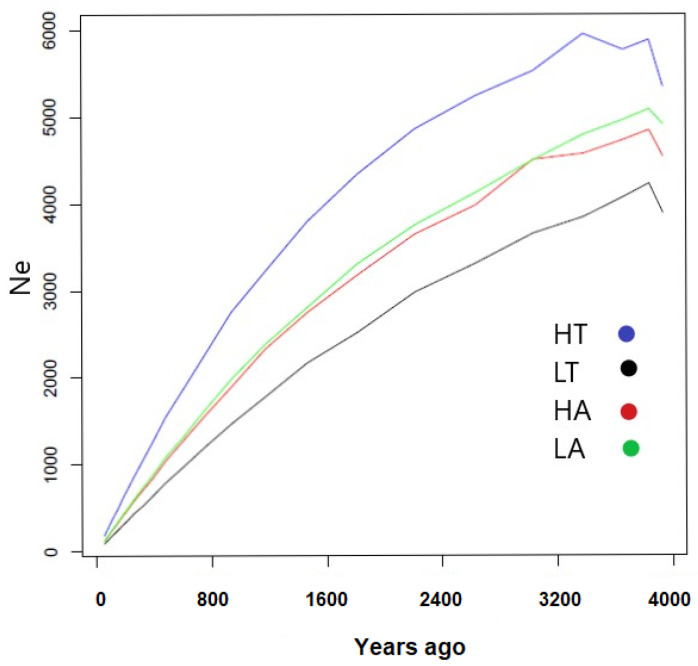
Estimated effective population sizes (*N_e_*) in Carpatina populations over the past 4000 years.

The overall low levels of LD suggested a high historical *N_e_* for Carpatina. Figure 3 and Table S2 show a declining trend of *N_e_* for the four groups across the past 4000 years. Globally, the four populations displayed the same trend of decreasing effective population size, which mirrors their shared ancestry. Between the two extreme *N_e_* estimates, i.e., the oldest and the most recent, we observed an almost 50–100-fold decline in *N_e_*, with the lowest being 92 (LT) (Figure 3).

## 4. Discussion

Goats are one of the major livestock species across the world and are of particular relevance for low-income farmers [27,28,29]. Overall, goats are hardy and require little input to be maintained [27]. While most goat breeds are local and characterized by relatively small population sizes, goats are also bred industrially for the production of milk, e.g., the Saanen breed [30,31]. The combination of their hardiness and suitability for selection in industrial settings makes them an ideal livestock species to address issues around climate adaptation [32,33]. Consequently, establishing baseline summary statistics that characterize goat breeds around the world is critical so their genetic variation can be monitored as these breeds face current and future environmental changes and are selected for traits of commercial value [9]. The genetic diversity estimates observed in our study are in accordance with the reported values for some South American and European goat populations, such as the Argentinian Angora goats (*H_E_* = 0.397, *H_o_* = 0.417) [14], Spanish goats (*H_E_* ranged between 0.29 and 0.42 and *H_o_* ranged between 0.28 and 0.41) [16] and Italian goats (*H_E_* ranged between 0.37 and 0.41 and *H_o_* ranged between 0.36 and 0.41) [13]. Our results are also consistent with those obtained for a limited number of Carpatina goats (N = 14) by Colli et al. [9], supporting the view that Carpatina harbor relatively high levels of genetic variation in comparison to other European and American goats.

The SNP-based estimates of genetic variation are, however, lower than those obtained using microsatellite markers, e.g., Chinese goat breeds (*H_E_* between 0.6433 and 0.806) [34,35], Malaysian goats (*H_E_* between 0.51 and 0.66) [36] and Canary Island goats (*H_E_* between 0.287 and 0.842). However, the differences observed are likely due to the nature of the mutation process in the two types of markers versus SNPs, and to the different technical requirements of microsatellite vs. SNP scoring. Microsatellites undergo a rapid mutation process due to slippage of the DNA polymerase when cells are replicating, resulting in substitution rates that range between values as high as 10^−2^ and 10^−5^ [37], whereas SNP substitution rates are much lower, e.g., 2.5 × 10^−8^ [1]. Furthermore, while SNP polymorphisms present on the commercial arrays are chosen among those displaying a biallelic behavior, microsatellites are multiallelic and usually selected to present high levels of polymorphism, consequently resulting in higher heterozygosity figures in microsatellites than in SNPs [38,39,40,41].

Inbreeding strongly contributes to the manifestation of genetic diseases of a recessive nature on the one side, and to the reduction of adaptative potential of population through the loss of genetic variation on the other side [42]. By controlling inbreeding, we can limit the potential impact of deleterious alleles and the loss of additive genetic variance [43]; however, basal levels of genetic variation are necessary so the development of inbreeding can be tracked and mitigated. The development of genomic tools, such as the SNP array technology implemented here, enables us to obtain information about thousands of markers across the genome at a reduced price [44]. Romanian Carpatina goats present overall high levels of genetic variation. Furthermore, the lack of deviation between their observed and expected heterozygosities suggests that they largely represent a genetically healthy random mating population, as testified also by the inbreeding coefficient not significantly differing from zero.

The overall value of r^2^ across chromosomes for the HA and LA groups was 0.05, and for the HT and LT groups 0.07. The mean value for all four groups was 0.06, further reflecting little linkage disequilibrium, consistent with the historically large effective population size and large genetic variation recorded in this study. Our results are in concordance with [45] the Eghoria (r^2^ = 0.084) and Skopelos (r^2^ = 0.087) breeds. Our study also reveals the fact that the number of SNPs in LD tends to decrease with decreasing chromosome length. We found that the mean r^2^ from our study was lower than those observed in other goat breeds—0.11 [14] (South African, French and Argentinian Angora goats), 0.14 [46] (Canadian Alpine, Boer, La Mancha, Nubian, Saanen and Toggenburg goats and Australian Boer, Cashmere and Rangeland goats), 0.18 [47] (British Apline, Saanen and Toggenburg goats)—and higher than in Chinese goat populations (0.02) [43] (Nanjiang, Qingeda, Arbas Cahsmere, Jining Grey, Louping Yellow and Guangfeng goats).

The pairwise divergence estimated with *F_st_* demonstrated that the four nuclei of Romanian Carpatina actually represent one single group without population structure, a view further supported by the partitioning of genetic variance in AMOVA analysis. The *F_st_* values scored here are substantially lower than those obtained by other authors in other breeds [14,16,48,49], and in line with those obtained for Romanian goats using 40 SNPs [4] and using 27 SNPs genotyped across eight European goat breeds [50]. Our observed lack of population structure within Carpatina also reflected the results of the SNP array data by Colli et al. [9], which shows that for the limited dataset they had (14 Carpatina individuals), the Admixture analyses did not render population structure within the breed. The only instance of some population structure reported for Carpatina is for Polish samples by Kaweçka et al. [51], who analyzed 249 animals from 14 farms using microsatellites. In their study, they argued for the identification of two groups of animals with one group largely represented by animals from one farm. Furthermore, their identification of two groups in the data is based on the use of Structure Harvester, which cannot assess whether one group (cluster) in the data is the optimal partition solution. As shown in the Kaweçka et al. [51] delta likelihood plot, a clustering solution with only one cluster is more likely than any of the other partitions suggested by the authors.

Transhumance is a traditional pastoral practice involving the seasonal movement of livestock, typically sheep and goats, between lowland and upland areas in search of better grazing and climate conditions [52]. This age-old method enables herders to optimize the use of available resources and respond to changing environmental conditions throughout the year. In Romania, transhumance has deep roots and is a crucial aspect of the country’s rural heritage [52], with the practice centered on the Carpathian Mountains, a vast and diverse range that spans much of the country. During the warmer months, shepherds guide their flocks, including Carpatina goats, from the lowland pastures to the alpine meadows at higher elevations. This migration is driven by the need for abundant forage and suitable conditions during the summer months, while in winter, herds return to lower altitudes where milder temperatures and more accessible food sources prevail. Carpatina’s ability to thrive in both lowland and highland environments is a testament to the breed’s adaptability.

However, Carpatina herds from different regions across Romania come in contact with each other during transhumance when animals are brought up to higher altitudes. During that period, there is an increased potential for interbreeding between herds. This intermingling is likely one of the main sources preventing the differentiation between groups of Carpatina goats.

Transhumance can also lead to breeding between Carpatina goats and locally occurring goats from other breeds. Efforts are being made to address these concerns and preserve the integrity of the Carpatina goat breed by avoiding its genetic erosion. Conservation programs and sustainable herding practices aim to maintain the distinct characteristics of the breed while allowing for the continuation of transhumance traditions. By striking a balance between preserving genetic diversity and sustaining traditional practices, Romania seeks to ensure the long-term viability of the Carpatina goat and the cultural heritage associated with transhumance in the Carpathian Mountains.

The effective population size is one of the most informative parameters in population genetics, because it reflects important aspects of the breeding process within populations and the impact of genetic drift, both important for conservation [53]. The patterns of N_e_ observed in Carpatina are consistent with the decline in effective population size observed in other goat breeds [9]. Recent studies by Alberto et al. (2018) and Colli et al. (2018) have shown that at the time when domestication occurred (i.e., 10,500–9900 years ago), goats had a larger effective population, which has been declining throughout time, and in particular in the recent past [9].

This decline is on one hand representative of the continuous selection since the start of the domestication period in goats [1] to obtain desirable phenotypes. Nevertheless, the recent strong decline in *N_e_* is worrisome, as modern breeding practices are likely to lead to the maintenance of low *N_e_* values for long periods of time, thus resulting in the loss of genetic variation. The *N_e_* estimates at 13 generations (52 years) ago of 185 (HT) and 92 (LT) put the *N_e_* values of the breed well below the threshold of 500, which is considered the lowest value necessary for populations to avoid losing evolutionary relevant genetic variation [54,55,56]. Furthermore, a critical *N_e_* of 100 has been suggested as the lowest threshold to maintain sustainable animal production while preserving genetic diversity [46,57].

## 5. Conclusions

This study of Romanian Carpatina goats overall presents a scenario of low impact of inbreeding on the current breed, with the occurrence of an ongoing gene flow between the flocks throughout the country thanks to the exchange of breeding individuals. This is achieved through the exchange of goats in animal markets and also via transhumance, an animal keeping method still used in Romania, which brings animals together over long periods of time, thus enabling mating between goats from different farms which would not be possible in industrial farming systems. Nevertheless, the trend in the effective population size in Carpatina is a concern, as it shows a strong decline in the last hundred years, resembling the general trend observed in several goat populations around the world [9]. Considering climate change and the genetic erosion that it may cause at the species and breed level, it is critical that the genetic variation of Carpatina is monitored to ensure it is not reduced further, which would potentially result in the loss of yet unknown adaptive variants.

## Figures and Tables

**Table 1:** Summary of sample code, sample size and sampling area of Carpatina goat.

Sample Code	Sample Size (*n*)	Sampling Area (County)	AAT **/ALT ***
HA	22	SV, SB, CV, BV, AG *	355 m–1241 m
LA	22	NT, BR, TL, CT, OT, TR	0 m–188 m
HT	33	BR, TL, CT, TR	12 °C–13.1 °C
LT	17	CV, BV	7.1 °C–7.6 °C

* SV = Suceava, SB = Sibiu, CV = Covasna, BV = Brasov, AG = Arges, NT = Neamt, BR = Braila, TL = Tulcea, CT = Constanta, OT = Olt, TR = Teleorman. ** Average annual temperature. *** Altitude.

**Table 2:** Measures of genetic diversity, sample size (*n*), average relatedness (PI_HAT), minor allele frequency (MAF), expected heterozygosity (*H_E_*), observed heterozygosity (*H_o_*) and inbreeding coefficient (*F_IS_*) amongst the studied goat populations.

Group Acronym	*n*	PI-HAT	MAF	*H_o_*	*H_E_*	*F_IS_*
HA	22	0.007	0.331	0.416	0.416	0.021
LA	22	0.004	0.333	0.419	0.418	0.019
HT	33	0.008	0.335	0.418	0.420	0.020
LT	17	0.007	0.328	0.416	0.413	0.022
Overall	67	0.007	0.332	0.418	0.418	0.021

**Table 3:** Pairwise *F_st_* values (below diagonal) between the four groups of Carpatina goats’ corresponding *p*-values (above the diagonal).

Group Symbol	HA	LA	HT	LT
HA		0.000	0.000	0.982
LA	0.007		0.991	0.000
HT	0.006	0.000		0.000
LT	0.000	0.006	0.005	

## Data Availability

The data presented in this study are available on request from the corresponding author. The data are not publicly available because the data will be used in future research.

## Supplementary Materials

The following supporting information can be downloaded at: https://www.mdpi.com/article/10.3390/ani14040560/s1, Table S1: Linkage disequilibrium r^2^ and SNPs number per chromosome in the studied four groups. Table S2: The declining trend of *N_e_* for the four Carpatina studied groups across the past 1000 generations.
