# Supplementary material for: Analysis of Genetic Diversity in Romanian Carpatina Goats Using SNP Genotyping Data
Source: Animals (Basel). 2024 Feb 7;14(4):560. doi: 10.3390/ani14040560 (PMC10886219; doi:10.3390/ani14040560)
Supplement: Supplementary file 1 [file animals-14-00560-s001.zip › animals-2799063-supplementary.pdf]

Table S1. Linkage disequilibrium r^2 and SNPs number per chromosome in the studied four groups.

CHR	HA		HT		LA		LT	
	r^2	SNPs number	r^2	SNPs number	r^2	SNPs number	r^2	SNPs number
1	0,0632	28494	0,0500	28530	0,0630	28530	0,0813	28404
2	0,0615	24534	0,0453	24517	0,0604	24481	0,0744	24499
3	0,0637	20368	0,0462	20421	0,0602	20367	0,0793	20295
4	0,0625	20961	0,0473	20997	0,0598	20943	0,0802	20942
5	0,0625	19566	0,0455	19584	0,0587	19494	0,0745	19440
6	0,0637	20601	0,0466	20583	0,0606	20493	0,0765	20510
7	0,0650	18914	0,0487	18963	0,0628	18968	0,0778	18896
8	0,0615	20412	0,0461	20412	0,0605	20395	0,0784	20357
9	0,0593	16587	0,0442	16534	0,0574	16552	0,0746	16551
10	0,0627	18288	0,0463	18306	0,0612	18306	0,0797	18234
11	0,0672	18638	0,0617	18675	0,0617	18675	0,0814	18578
12	0,0715	14976	0,0590	15013	0,0710	15011	0,0869	14937
13	0,0632	14221	0,0466	14292	0,0613	14132	0,0807	14186
14	0,0622	16526	0,0476	16596	0,0630	16596	0,0796	16451
15	0,0604	13986	0,0455	14022	0,0599	14004	0,0763	13949
16	0,0620	13788	0,0457	13841	0,0594	13806	0,0774	13751
17	0,0603	12744	0,0450	12762	0,0601	12717	0,0743	12743
18	0,0678	10934	0,0497	10971	0,0643	10935	0,0843	10898
19	0,0598	10605	0,0442	10656	0,0595	10620	0,0795	10549
20	0,0619	13005	0,0450	13023	0,0595	12987	0,0744	12969
21	0,0605	12375	0,0476	12375	0,0612	12303	0,0771	12286
22	0,0675	10215	0,0458	10215	0,0616	10179	0,0804	10163
23	0,0620	9018	0,0460	9018	0,0598	8982	0,0750	8965
24	0,0636	11511	0,0483	11529	0,0619	11529	0,0781	11457
25	0,0596	7389	0,0431	7407	0,0587	7407	0,0776	7336
26	0,0616	9063	0,0476	9063	0,0619	9063	0,0798	9027
27	0,0629	8035	0,0448	8073	0,0607	8073	0,0797	8073
28	0,0595	8010	0,0411	8028	0,0568	8010	0,0766	7938
29	0,0594	8307	0,0414	8343	0,0559	8289	0,0736	8253
**Average**	**0.0627**		**0.0469**		**0.607**		**0.0782**	

Table S2. The declining trend of N_e for the four Carpatina studied groups across the past 1000 generations.

	**high alt**	**high temp**	**low alt**	**low temp**
**GenAgo**	**Ne**	**Ne**	**Ne**	**Ne**
983	4537	5340	4907	3887
959	4841	5879	5080	4226
914	4726	5764	4956	4068
844	4570	5947	4789	3841
758	4503	5522	4497	3654
658	3979	5236	4123	3310
553	3648	4857	3754	2981
453	3177	4336	3306	2517
366	2747	3793	2804	2168
293	2328	3221	2381	1779
234	1893	2752	1982	1468
187	1555	2253	1619	1197
150	1274	1863	1314	972
120	1048	1551	1090	795
98	850	1271	888	647
80	709	1051	737	527
65	588	868	607	443
54	487	727	502	365
45	411	615	425	307
38	345	509	356	260
32	292	438	303	220
27	250	375	258	188
23	215	321	221	162
20	186	279	191	139
17	161	241	165	121
15	141	211	143	105
13	123	185	125	92
